# Case Report: Pneumocephalus after labor epidural anesthesia

**DOI:** 10.12688/f1000research.4693.1

**Published:** 2014-07-22

**Authors:** Beatriz Nistal-Nuño, Manuel Ángel Gómez-Ríos

**Affiliations:** 1 Department of Anaesthesiology and Perioperative Medicine, Complexo Hospitalario Universitario A Coruña (CHUAC), A Coruña, 15006, Spain

## Abstract

Lumbar epidural anesthesia is commonly used for labor analgesia. The 'loss-of- resistance' to air technique (LORA) is generally employed for recognition of the epidural space. One of the rare complications of this technique is pneumocephalus (PC). Here we describe the case of a parturient who developed a frontal headache when locating the epidural space using LORA. On the second day after epidural injection, the patient exhibited occipital headaches with gradual worsening. Computed tomography scans of the brain indicated PC. Following symptomatic treatment, our patient was discharged on the 13th day. We concluded that the amount of air used to identify the epidural space in LORA should be minimized, LORA should not be used after dural puncture and the use of saline avoids PC complications.

## Introduction

Lumbar epidural anesthesia is a commonly used technique for analgesia during labor. The complications often associated to this technique include unilateral analgesia, extended epidural blockade, unplanned puncture of the dura or of a blood vessel, post-puncture dural headache (PDPH), subdural blockade, placement of the catheter out of the epidural space and neurological complications ^1^. The ‘loss-of-resistance’ to air technique (LORA) is commonly employed for recognition of the epidural space. Nevertheless, one of its rare complications is pneumocephalus (PC) ^2^.

Cases of PC following neuroaxial anesthesia have been described either after an epidural using the LORA technique or after spinal anesthesia ^3, 4^. The development of PC after spinal anesthesia is exceptionally rare ^5, 6^. PC symptoms are difficult to distinguish from other complications of the epidural technique such as PDPH or neurotoxicity. Diagnosis relies on clinical impression and brain tomography (CT-scan) ^2^. Our goal is to report the case of a patient who presented PC following labor epidural anesthesia.

We attempted to approach the patient and the patient’s family to obtain informed consent for publication of this report, However we decided to abandon telephone contact after numerous unsuccessful attempts. The Institutional Review Board at Complexo Hospitalario Universitario A Coruna determined that approval of the case report was not required.

## Clinical case

A 34 year old healthy Caucasian parturient ASA II, G1P0 was admitted at 38 weeks of gestation. Her clinical history included no allergies to medications, no relevant family history, mild bicuspid aortic valve stenosis, cervical aortic arch and mild postductal coarctation of the aorta. She was not taking any medications. She presented occasional asymptomatic palpitations at rest (few seconds long). Her cardiopathy was compensated and well controlled during pregnancy.

At 4–5 cm of cervical dilatation, at the request of analgesia, a lumbar epidural was proposed for management of labor pain. The patient was monitored with ECG, SpO2, non-invasive blood pressure (NIBP) every 5 minutes and a peripheral vessel catheter was *in situ*. Puncture was performed at the L4–L5 interspace with a 18 G Tuohy epidural needle (Perifix ^®^ 401 Braun Germany) (80 mm/3¼" long) via the median approach with the patient in the sitting position. After locating the epidural space using the LORA technique (approximately 3 ml of air), the patient developed a sudden intense frontal headache. No neurological, haemodynamic changes or breathing symptoms were detected. The needle was withdrawn without cerebrospinal fluid (CSF) flashback. The cephalalgia improved gradually ceasing several minutes later. An epidural catheter was introduced afterwards (Perifix ^®^ 401 Braun Germany, close-end, three lateral holes) in the L3–L4 interspace with the patient in the sitting position, after locating the epidural space using loss of resistance to saline (LORS). We confirmed its appropriate location by checking that CSF had not been aspirated prior to local anesthetic injection. The test dose of 3 ml of bupivacaine 0.25% and epinephrine resulted negative. An initial bolus of 5 ml of bupivacaine 0.25% and 50 mcg of fentanyl was administered and a continuous infusion of ropivacaine 0.18% in addition to 1 mcg of fentanyl per ml was programmed at 7 ml/hour. This process was incident-free, being the epidural analgesia correct and labor with normal evolution.

**Figure 1.**
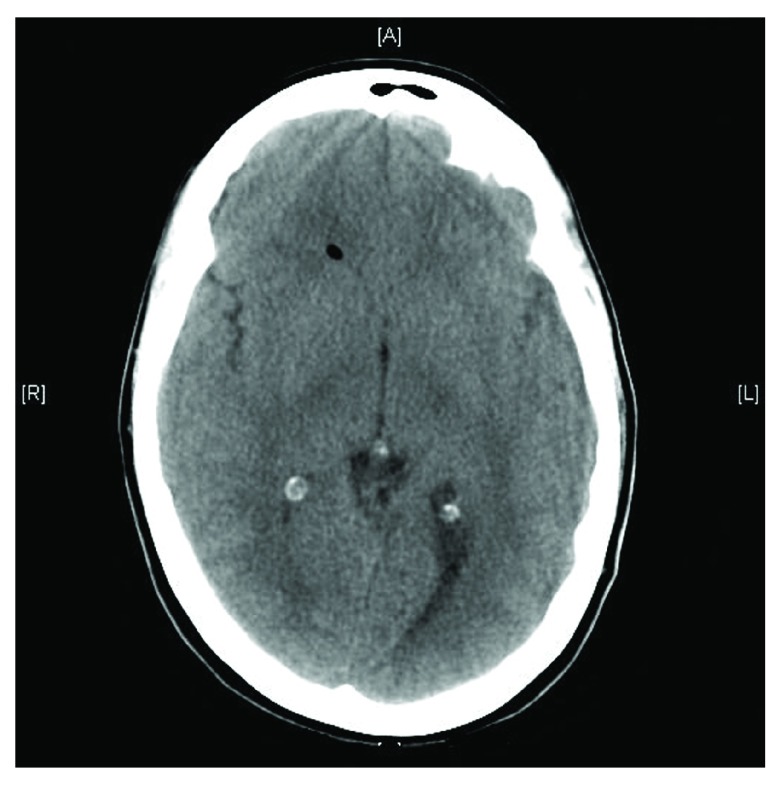
Axial CT scan shows the presence of a gas bubble in the ventricular system following dural puncture, in the right frontal cistern horn of the lateral ventricle.

**Figure 2.**
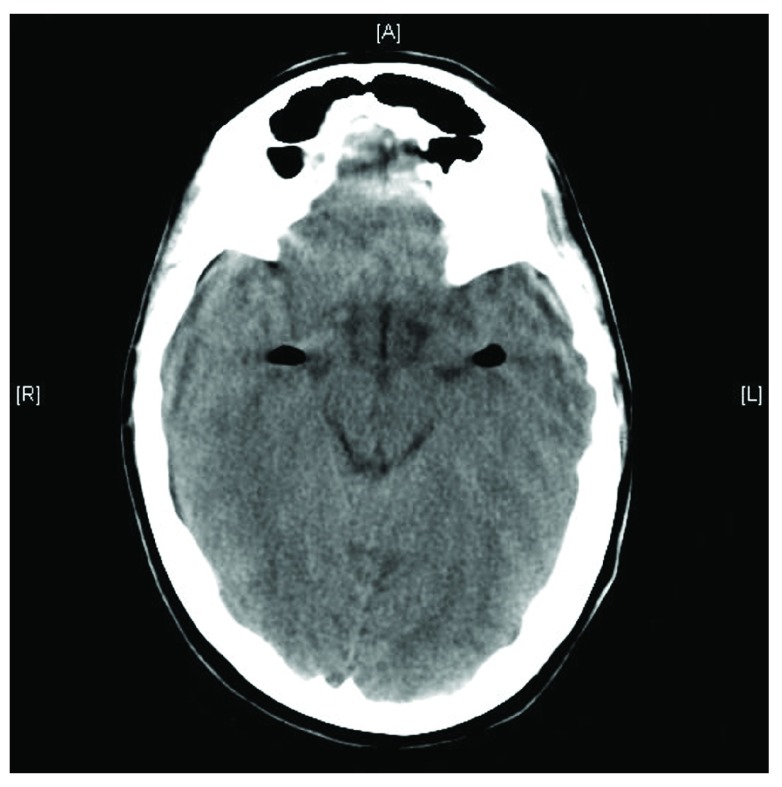
Axial CT scan shows the presence of a gas bubble in the ventricular system following dural puncture, in both temporal cistern horns.

On the second day postpartum, the patient presented occipital headaches which increased on standing, accompanied by tinnitus, nausea and vomiting, with gradual worsening. We found no alterations in the neurological examination. We decided conservative treatment with rest, NSAIDs, intravenous hyperhydration and 300 mg of oral caffeine twice daily, showing mild clinical improvement. However, on the 6th day after delivery, due to mild clinical worsening, and suspecting iatrogenic PC vs. PDPH, a brain CT-scan was performed. The CT scan revealed air in the temporal horns and right frontal horn of the lateral ventricle, showing a PC in the subarachnoid space and ventricles that was responsible for mild ventricular dilation ( Figure 1 and Figure 2). A subdural collection that could correspond to an inflammatory reaction was also observed. At all times the neurological examination was normal. We consulted the Neurosurgery Service, which considered the PC not significant and recommended to continue with the conservative treatment and repeat the CT in 48–72 hours or if neurological changes occurred.

Despite treatment, at the 8th day after puncture, the patient’s symptoms worsened at night with severe frontal headaches (Visual Analog Scale 6) and an increase in tinnitus. She was afebrile, the vital signs were unremarkable, and she had no focal neurological deficits. A control CT scan showed a mild regression of the PC in the ventricular system, but demonstrated persistence of the subdural collection. We decided to continue with the conservative treatment and performed control blood tests (routine hematological; liver and kidney function tests; serum electrolytes; coagulation profile).

On the 13 ^th^ day after delivery, the follow-up was uneventful, the patient showed no abnormalities at the neurological examination and her vital signs were normal. The patient was finally discharged from the anesthesiology department being completely asymptomatic.

## Discussion

PC is relatively common in neurosurgery ^7^ and neuroradiology ^8, 9^. It can be caused by trauma ^10^ or infections ^11, 12^. It may develop after lumbar puncture, epidural steroid injection, or Valsalva’s maneuver ^13– 15^. The development of PC after spinal or epidural anesthesia is extremely infrequent. The incidence of PC after epidural steroid injections or epidural anesthesia is unknown, and only few cases per year are described in the literature ^16, 17^. Most cases of PC due to epidural techniques have been associated with LORA, as described in our case report.

PC, an unusual consequence of evident or unnoticed accidental dural puncture ^3, 4, 18^, develops from the injection of air into the subarachnoid or subdural space and cranial migration ^19^. PC is not often followed by symptoms, but among those, headache is the most frequent ^20, 21^. The appearance of other symptoms, such as signs of space-occupying lesions (focal neurologic deficits including cranial nerve palsies ^19, 20^, or diverse motor signs) or augmented intracranial pressure and cardiovascular instability may develop depending on the spread and extent of intracranial air ^22^. Headache is caused by the fast brain motion resulted from air injection and meningeal irritation ^21^. Most cases consist of abrupt intense frontotemporal cephalea ^5, 23, 24^, as in our case study, having a premature beginning (same day) and commonly concluding within 5 days. It is exacerbated by motion and may not be alleviated by lying down ^2^. Roderick *et al.* outlined that 2 ml of air injected into the subarachnoid space was sufficient to provoke a symptomatic PC ^5^.

In case of PDPH, due to CSF outflow, the pain is exacerbated by sitting or standing and is alleviated by lying down, having a characteristic occipital, frontal and post-orbital situation. It happens more often 24 to 48 hours after dural puncture ^25^ and is longer lasting than in PC ^2^. Although there may be subtle clinical differences with PC, their symptoms usually are interchangeable so the differential diagnosis must be done through CT.

A number of techniques to find the epidural space have been defined ^26^. LORA and LORS are the most common methods used ^2^. Potential inconveniences of using saline comprise the difficulty to ascertain a meningeal puncture ^27^. On the other hand, if air is forced quickly by digital pressure, false positives may result, or gas embolism, subcutaneous emphysema ^28^ or multiradicular syndromes ^29^.

Accidental dural puncture is not always evident, as also shown in our case. Okell and Springge ^30^ described a 0.6% incidence of dural punctures in epidural anesthesia. These punctures were acknowledged by the loss of CSF, by aspiration through the catheter, by hypotension after injection of a test dose, and retrospectively. Hardy ^31^ reported that an epidural catheter cannot easily be passed through the dura, but the arachnoid can be penetrated smoothly. The author deduced that when a catheter goes into the subarachnoid space it is due to its initial subdural placement and movement to the arachnoid, as the first hole in the dura yields migration of the catheter ^2^. Air introduced into the subdural space is more painful than in the subarachnoid space and reaches rapidly the head, because of its low pressure and diminished capacitance ^32, 33^.

These findings explain the sequence in our case. We found no evidence of dural puncture with CSF flashback with the needle insertion at the L4-L5 interspace, but the dura had already been penetrated, allowing the passage of air likely into the subdural space and causing the abrupt headache. Despite our uncertainty regarding dural puncture, we correctly avoided LORA and used LORS for the following attempt to localize the epidural space.

In our case, PC was diagnosed from the CT scan. While subdural space is not straightly connected to the subarachnoid space ^34^, it is annexed to the floor of the third ventricle in the cranial cavity from the lower border of the second sacral vertebra ^35, 36^.

The treatment of PC consists on administration of 40–100% oxygen in the supine position ^37^. This is to favor the reabsorption of intracranial air by intensifying the diffusion concentration gradient for nitrogen between the air collection and the surrounding cerebral tissue ^38^. Nitrous oxide should be avoided to prevent the expansion of PC ^39^. In addition, we should administer aggressive hydration, caffeine, or analgesics ^40, 41^. Epidural infusion or blood patch have no effect on PC ^3, 19^.

There is usually reabsorption of the air within 3–5 days from the epidural injection and patients commonly improve without any neurologic abnormalities. Our patient was discharged after clinical-radiologic resolution on the 13th day. If tension PC occurs, a neurosurgical emergency treatment may be necessary ^21, 23, 42^.

In conclusion, the way to minimize the likelihood of PC when performing epidural block with the loss of resistance technique is to use saline instead of air ^43, 44^. When air is used, we should minimize its extent. In addition, LORA should not be used after dural puncture.

After epidural block, and particularly when dural puncture is performed, the patient should be monitored carefully. We should identify that the headache from PC after epidural anesthesia occurs commonly immediately after puncture. Likewise, we should recognize that symptoms of PC are similar to those of PDPH and that a differential diagnosis is established by imaging techniques. Lastly, we should be able to assess that PC may be spontaneously absorbed, managed with symptomatic treatment.

## Consent

After numerous unsuccessful attempts to contact the patient and the patient’s family to obtain informed written consent for publication of this report, the Institutional Review Board at our Institution determined that approval of the case report was not required.
