# Supplementary material for: Case Report: Pneumocephalus after labor epidural anesthesia
Source: F1000Res. 2014 Jul 22;3:166. [Version 1] doi: 10.12688/f1000research.4693.1 (PMC4156026; doi:10.12688/f1000research.4693.1)
Supplement: Peer review history

# Peer review: Referee response for version 1

**DOI:** 10.5256/f1000research.5014.r5559

**Published:** 2014-08-14

**Authors:** Jean Eloy

**Affiliations:** 1 Department of Anesthesiology, Rutgers New Jersey Medical Center, Newark, NJ, USA

The case report: “Pneumocephalus after epidural anesthesia” is a well written manuscript. The Hypothesis is sound and the originality of the manuscript evident. The report is also relevant to clinical practice, shows appropriate methodology and is properly referenced. However, in the introduction the author states that the development of PC after spinal anesthesia is exceptionally rare - which it is not. I would omit the word exceptionally from the manuscript, as PC is often confused with post dural puncture headache and is often underreported.

As the author mentioned in the conclusion section, using loss of resistance to saline instead of air is a way to minimize the incidence of PC. However the author does not expand on which technique is used the most by practitioners and taught in residency programs.  Although this case report is hardly the definitive paper on Pneumocephalus after epidural analgesia, it certainly offers guidance and raises awareness on its incidence.

1. From the case report, the author report sudden onset of frontal headache that improved within several minutes. There was no mention in the case report or the discussion on when the epidural catheter was removed and the temporal relationship with the patient occipital headaches.
2. The signs and symptoms of PDPH and PC are the same. Are the symptoms reported by the   author the results of PDPH or PC?  This has not been made

I have read this submission. I believe that I have an appropriate level of expertise to confirm that it is of an acceptable scientific standard.

# Peer review: Referee response for version 1

**DOI:** 10.5256/f1000research.5014.r5543

**Published:** 2014-08-04

**Authors:** Jean Charchaflieh, Ivan Velickovic

**Affiliations:** 1 Department of Anesthesiology, Yale University, New Haven, CT, USA; 2 SUNY Downstate Medical Center, Brooklyn, USA

The authors provided an excellent description of a case of pneumocephalus (PC) after epidural catheterization, without evident dural puncture, with very informative discussion of the pathophysiology, symptomatology and management. The authors discussed briefly some of the advantages and disadvantages of using loss of resistance to air (LORA) versus loss of resistance to saline (LORS) in identifying the epidural space. The authors provided three recommendations for the prevention of PC after epidural catheterization, which are not necessarily supported by empirical evidence or consensus of practitioners. The three recommendations are:

**1.** Use LORS instead of LORA to minimize the likelihood of PC after epidural catheterization.

This recommendation is not supported by empiric evidence or consensus of practitioners. Indeed one of the cited references in support of this recommendation indicates that all three reported cases of PC occurred after LORS; and that the general practice in UK is about 50% LORS and 50% LORA. ^43 in main article^

^^

As the authors noted, one of the potential disadvantages of LORS is clouding the certainty of the occurrence of dural puncture. ^27 in main article^

Another potential disadvantage of LORS is creating a pocket of fluid in front of ligamentum flavum that allows placing the epidural catheter in front of ligamentum flavum instead of the epidural space.

The argument that LORS minimizes the likelihood of PC after epidural catherization is not clear cut and not the overriding consideration in choosing LORA vs. LORS.

**2.** Minimize the amount of air when using LORA.

When using LORA, most practitioners would argue in favor of limiting the amount of injected air to 1-2 ml, and against the use of multiple confirmatory injections of air into the epidural space. However as the presented case demonstrate, PC can occur even when performing LORA with approximately 3 ml of air and without evident dural puncture.

**3**. LORA should not be used after dural puncture.

This recommendation is not supported by empiric evidence and not universally endorsed by practitioners. As noted above, LORA can be more advantageous than LORS at detecting dural puncture. As for the immediate management of dural puncture, interventions can vary from inserting a catheter into the intrathecal space to reattempting the epidural catheterization at a different lumbar level. No specific technique has proven an advantage or disadvantage regarding the risk of PC. Indeed, as the presented case demonstrates, PC can occur even when LORS is used after initial LORA without excessive amount of air and without evident dural puncture.

It is worth noting that post-dural puncture headache (PDPH) can co-exist and outlast headache due to PC. One of the referees (IV) reported a case of PC for 3 days, followed by resolution of PC and subsequent occurrence of typical PDPH, which resolved with treatment with epidural blood patch ( Velickovic & Pavlik, 2007).

Overall, the authors presented a well-balanced case report and discussion, which can serve as a handy review for diagnosis and management of PC after epidural catheterization.

We have read this submission. We believe that we have an appropriate level of expertise to confirm that it is of an acceptable scientific standard.

# Peer review: Referee response for version 1

**DOI:** 10.5256/f1000research.5014.r5538

**Published:** 2014-07-29

**Authors:** Richard Smiley

**Affiliations:** 1 Department of Anesthesiology, Columbia University, New York, NY, USA

The authors have reported an interesting case of headache after a lumbar epidural procedure. There is value in reminding anesthesiologists of the potential for pneumocephalus (PC) when air is used for the loss-of-resistance procedure. Their description makes it clear that the patient developed an almost immediate headache, which resolved rather quickly. This was followed by a more chronic, at least partially postural headache starting day 2 after the procedure. While the initial headache was almost certainly due to air injection into the subdural or subarachnoid space and a resulting PC, the subsequent headache may well have been a more classic post-dural puncture headache (PDPH), perhaps with some residual from the PC, given its time course (starting day 2, continuing for the better part of two weeks, which is later and longer than most PC headaches, but exactly what one might expect from a PDPH).  

The authors mention that PC headaches typically last less than 5 days (the few we have seen have tended to last hours not days). A bit more discussion of this possibility/probability could be in the manuscript. It is possible, especially when one considers that the neurosurgeons considered the PC “”not significant,” that the patient could have been treated with a epidural blood patch to relieve her headache and shorten her hospital stay. In general, PC headaches are not as obviously postural as PDPH, so while certainly symptoms overlap, I am not sure it is fair for the authors to state that the symptoms are “interchangeable.”

The authors suggest that because no CSF was seen with the initial placement the Tuohy needle may have been in the subdural space, and give an anatomic explanation of how air gets form the subdural to the intracerebral subarachnoid space; this is a possible explanation, but it is also possible that the air was injected subarachnoid but the needle withdrawn before CSF was appreciated. It is interesting that Roderick, in 1985 (ref 5 in this case report) reported a PC after injection of ~ 2ml air during attempted spinal anesthesia; in that case too there were problems obtaining CSF; perhaps they were subdural.

There are some unimportant issues with English usage that do not detract from understanding the main points of the case report.

I am not entirely sure I understand the mechanism of headache from PC as described in the case report.  The authors cite “fast brain motion” and “meningeal irritation.”  Any brain movement must be short-lived, explaining only the immediate, initial headache, and I am not sure how air is much of an “irritant.”  Unless the air is of a very significant volume, it seems likely that it has quite small effects after a few hours or a day or two; hence my suspicion this case is more a PDPH than  PC headache.

I have read this submission. I believe that I have an appropriate level of expertise to confirm that it is of an acceptable scientific standard, however I have significant reservations, as outlined above.
